# Maintenance therapy with histamine plus IL-2 induces a striking expansion of two CD56^bright^ NK cell subpopulations in patients with acute myeloid leukemia and supports their activation

**DOI:** 10.18632/oncotarget.10191

**Published:** 2016-06-21

**Authors:** Angélica Cuapio, Mirte Post, Sabine Cerny-Reiterer, Karoline V. Gleixner, Gabriele Stefanzl, Jose Basilio, Susanne Herndlhofer, Wolfgang R. Sperr, Nicolaas H.C. Brons, Emilio Casanova, Jacques Zimmer, Peter Valent, Erhard Hofer

**Affiliations:** ^1^ Department of Vascular Biology and Thrombosis Research, Medical University of Vienna, Vienna, Austria; ^2^ Department of Internal Medicine I, Division of Hematology & Hemostaseology, Medical University of Vienna, Vienna, Austria; ^3^ Ludwig Boltzmann Cluster Oncology, Medical University of Vienna, Vienna, Austria; ^4^ National Core Facility Cytometry, Luxembourg Institute of Health, Esch-sur-Alzette, Luxembourg; ^5^ Ludwig Boltzmann Institute of Cancer Research, Vienna, Austria; ^6^ Institute of Pharmacology, Center of Physiology and Pharmacology, Comprehensive Cancer Center, Medical University of Vienna, Vienna, Austria; ^7^ Department of Infection and Immunity, Luxembourg Institute of Health, Esch-sur-Alzette, Luxembourg

**Keywords:** natural killer cells, CD56^bright^, CD56^dim^, histamine, IL-2

## Abstract

Histamine dihydrochloride (HDC) plus IL-2 has been proposed as a novel maintenance-immunotherapy in acute myeloid leukemia (AML). We analyzed the immunophenotype and function of natural killer (NK) cells in blood of AML patients treated after chemotherapy with HDC plus IL-2. The treatment caused a striking expansion of CD56^bright^CD16^neg^ and CD56^bright^CD16^low^ NK cell subpopulations. A reduced NK cell fraction recovered and high proportions of cells expressed the activating receptors NKG2D, NKp30, and NKp46. Concomitantly, KIR-expressing NK cells were reduced and NK cells with inhibitory NKG2A/CD94 receptors increased beyond normal levels. In addition, the immunotherapy-induced NK cells exhibited high capacity to produce IFN-γ and to degranulate. Furthermore, we provide evidence from subsequent *in vitro* studies that this is caused in part by direct effects of IL-2 on the CD56^bright^ cells. IL-2 specifically induced proliferation of both CD56^bright^ subpopulations, but not of CD56^dim^ cells. It further preserved the expression of activating receptors and the capacity to produce IFN-γ and to degranulate. These data suggest that therapy with HDC plus IL-2 supports the reconstitution of a deficient NK cell fraction through the specific amplification of CD56^bright^ NK cells giving rise to a functional NK cell compartment with high potential to combat leukemic disease.

## INTRODUCTION

Immune surveillance by natural killer (NK) cells and T lymphocytes plays a fundamental role in controlling residual leukemic cells after chemotherapy or after stem cell transplantation in acute myeloid leukemia (AML). To eliminate such residual AML cells, NK cells and T cells use their cytotoxic and immunomodulatory properties, i.e. anti-leukemic cytotoxicity and cytokine secretion [1, 2]. Several lines of evidence suggest that the risk of relapse and thus poor prognosis in AML patients are partially due to functional deficiencies of NK cells, mainly related to a low expression of activating receptors and impaired cytotoxic activity [3–5].

Treatment options for AML include chemotherapy and/or stem cell transplantation (SCT) [6, 7]. For permanent eradication of the disease, T and NK cell-mediated graft versus leukemia effects after hematopoietic SCT are critical [8, 9]. In particular, allogeneic NK cells possess a potent effect against leukemia cells with both matched-, and mismatched-HLA combinations [10, 11] and furthermore, an efficient and rapid NK cell recovery after transplant is associated with an improved clinical outcome [12]. In the context of SCT, NK cell alloreactivity supposedly eliminates residual AML cells and thereby decreases the risk of relapse [13].

In a different approach to reduce AML relapse risk, treatment with IL-2 after successful chemotherapy has been proposed, with the idea to trigger the anti-leukemic properties of NK and T cells. This was evaluated in several clinical studies [14–17], but the outcome showed that monotherapy with IL-2 has no significant clinical efficacy in AML and this treatment modality is therefore no longer used. However, in 2007, a phase III clinical trial studying more than 300 AML patients reported that post-consolidation immunotherapy with histamine dihydrochloride (HDC) and low dose IL-2 reduces the relapse rate [18]. The rationale for using HDC is to inhibit the formation and release of reactive oxygen species (ROS) produced by myeloid cells [19, 20]. In this way, NK and T cells are protected from inactivation by ROS [21, 22] and furthermore, in a synergizing fashion, IL-2 should activate and enhance the immunostimulatory and cytotoxic capability of NK and T cells against leukemic cells mainly by inducing the release of cytokines and cytolytic granules.

In human peripheral blood, NK cells constitute 10 to 15% of the lymphocytes. A balance of inhibitory and activating signals regulates NK cell activation after receptor-mediated identification of ligands expressed by target cells [23]. Based on the expression of CD56, two main subpopulations of NK cells have been identified, the CD56^dim^ and the CD56^bright^ NK cells [24–26]. The CD56^dim^ subset represents approximately 90% of the peripheral blood NK cells and is known as the cytotoxic subpopulation due to its high capacity for natural cytotoxicity and CD16-mediated antibody-dependent cell cytotoxicity (ADCC). The remaining 10% comprises CD56^bright^ NK cells, described as a preferentially immunoregulatory and cytokine-secreting subset.

In this study, we investigated the effects of maintenance therapy with HDC plus low dose IL-2 on the proportion, phenotype and functional capacities of NK cells in AML patients displaying remission after chemotherapy. Our results show preferential and potentially beneficial effects of the treatment 1) on the expansion of two subsets of immunomodulatory CD56^bright^ NK cells distinguished by CD16 expression, 2) on the normalization of NK cell numbers, 3) on high expression levels of activating receptors and 4) on high capacity to produce IFN-γ and to degranulate. These effects on the pool of responsive NK cells suggest that the therapy increases the potential for the NK cell compartment to trigger anti-leukemic immune mechanisms in AML.

## RESULTS

### HDC plus IL-2 causes a striking expansion of two CD56^bright^ NK cell subsets in AML patients

**Figure 1:**
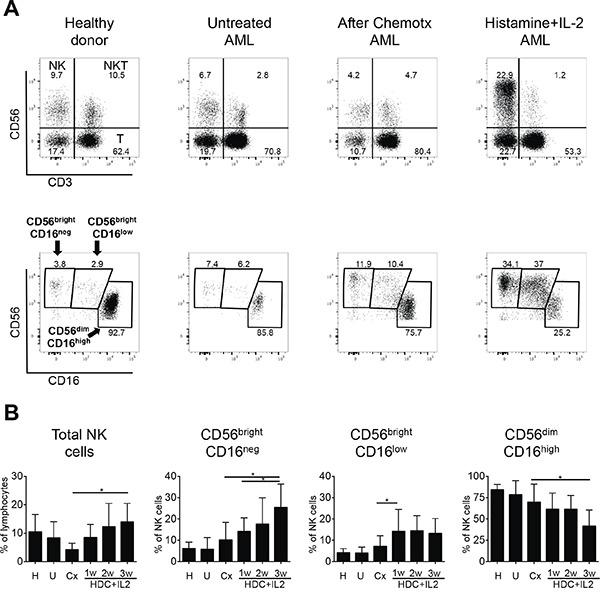
Distribution of peripheral NK cells in healthy donors and AML patients **A.** Representative dot plots of PBMC are shown for healthy donors and AML patients either untreated at time of diagnosis, after chemotherapy in remission, or treated with histamine dihydrochloride (HDC) plus IL-2. PBMC were stained with anti-CD3, -CD56 and -CD16 monoclonal antibodies and analyzed by flow cytometry. NK cells were identified after gating on lymphocytes as CD56^pos^CD3^neg^ cells (upper panels). The different NK cell subsets were gated based on the differential expression of CD56 and CD16 as CD56^bright^CD16^neg^, CD56^bright^CD16^low^ and CD56^dim^CD16^high^ (lower panels). **B.** Percentages of total NK cells within the lymphocyte fraction and of single NK cell subsets within the CD56^pos^CD3^neg^ NK cell fraction from 48 healthy donors (H), 11 untreated AML patients (U), 9 AML patients after chemotherapy (Cx) and AML patients undergoing additional HDC plus IL-2 therapy after 1, 2 and 3 weeks (w) of treatment with HDC plus IL-2 (1w: n=9; 2w: n=5; 3w: n=6) are shown as mean values +/− SD. *p<0.05

In initial experiments we examined the classical CD56^bright^ and CD56^dim^ NK cell subpopulations in healthy donors, untreated AML patients at time of diagnosis, AML patients in remission following chemotherapy and AML patients following treatment with HDC plus low dose IL-2. A closer analysis of the NK cells in the samples obtained from AML patients treated with HDC plus IL-2 revealed an increase of two distinct subsets of CD56^bright^ NK cells: CD56^bright^CD16^neg^ and CD56^bright^CD16^low^ cells (see representative dot plots in Figure 1A). The CD56^bright^CD16^low^ NK cells were present in healthy individuals only at very low numbers. When we tested expression of the markers KIR, NKG2A and CD57 in this subset, intermediate expression levels between the CD56^bright^CD16^neg^ and CD56^dim^CD16^high^ subsets were obtained supporting that the CD56^bright^CD16^low^ NK cells constitute an intermediate maturation stage of NK cells (see Suppl. Fig. 2). In the subsequent analyses, we therefore focused on both CD56^bright^ NK cell subsets as well as on the CD56^dim^CD16^high^ cells.

In AML patients in remission after chemotherapy, the proportion of NK cells in the lymphocyte fraction appeared to be reduced in comparison to healthy donors. However, the proportion of NK cells in patients treated with HDC plus IL-2 after chemotherapy increased significantly over three weeks (Figure 1B, left panel). Furthermore, within the NK cell fraction, the proportion of the two CD56^bright^ subsets clearly expanded significantly over one to three weeks of treatment (about three- and two-fold after three weeks for the CD56^bright^CD16^neg^ and CD56^dim^CD16^high^ subsets, respectively), whereas the proportion of CD56^dim^CD16^high^ NK cells displayed a concomitant decrease (Figure 1B, middle and right panels). A further analysis of absolute numbers of NK cells in peripheral blood displayed strongly increased cell numbers for total NK cells after treatment, reaching levels in the range of healthy donors (Suppl. Fig.1, left panel). Within the NK cell fraction mainly the CD56^bright^CD16^neg^ subset and to some extent also the CD56^bright^CD16^low^ subset increased strongly in cell number (on average about 10- and 5-fold, respectively), whereas the number of CD56^dim^CD16^high^ NK cells seemed to increase only slightly, but this did not reach significance (Suppl. Figure 1, middle and right panels).

### The impaired expression of activating NK cell receptors in AML patients is normalized after chemotherapy and further maintained during HDC plus IL-2 treatment

**Figure 2:**
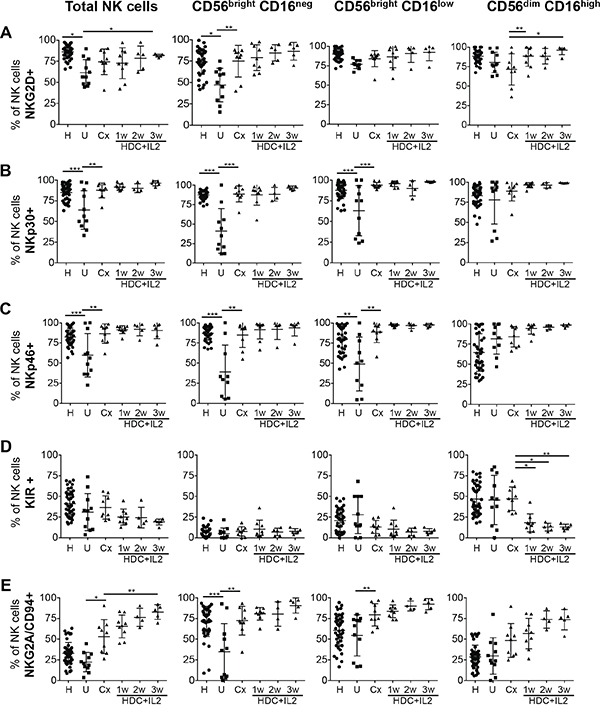
Expression profiles of activating and inhibitory receptors on total NK cells and NK cell subsets PBMC from healthy controls (H), untreated AML patients (U), patients after chemotherapy (Cx) and patients treated with HDC plus IL-2 for 1-3 w were used to analyze by flow cytometry the expression of the receptors NKG2D, NKp30, NKp46, KIR and NKG2A/CD94. on total NK cells and on single NK cell subsets. The percentages of cells positive for the specific markers were determined as the percentages of cells stained with the respective specific antibodies minus the percentages of cells stained with isotype-matched negative control antibodies. Mean values +/− SD of the percentages within the CD56^pos^CD3^neg^ NK cell fraction are shown. H: n=48; U: n=11; Cx: n=9; HDC+IL2 1w: n=9; 2w: n=5; 3w: n=6. *p<0.05, **p<0.01, ***p<0.001.

We next evaluated the expression of activating and inhibitory NK receptors on the different subsets of NK cells in untreated AML patients and patients after chemotherapy and HDC plus IL-2 treatment. It was intriguing that relative to healthy controls, the portion of NK cells expressing the activating NKG2D receptor and the natural cytotoxicity receptors (NCR) NKp30 and NKp46 were significantly reduced in untreated AML patients at the time of diagnosis. However, following chemotherapy the expression levels were again comparable to healthy donors and these levels were maintained during HDC plus IL-2 treatment (Figure 2A, 2B, 2C).

### Expression of KIR is reduced, but NKG2A/CD94 is increased beyond normal levels after HDC plus IL-2 therapy

To analyze the expression of KIR on NK cells, we used a pan-KIR antibody recognizing the inhibitory 2DL2 and 2DL3 as well as the activating 2DS2 and 2DS4 receptors. In contrast to NCRs and NKG2D, KIRs are generally not or minimally expressed on CD56^bright^ cells but are acquired late during NK cell maturation leading to high KIR expression only in the CD56^dim^CD16^high^ subset [27]. In healthy individuals, there is a gradual increase in the number of cells with KIR expression starting with the lowest level in the CD56^bright^CD16^neg^, intermediate levels in the CD56^bright^CD16^low^ and highest expression in the CD56^dim^CD16^high^ subset (Figure 2D and Suppl. Fig. 2).

When we analyzed KIR expression in untreated AML patients and patients after chemotherapy, no significant differences compared to healthy individuals were observed for total NK cells or any of the subsets. However, in AML patients treated with HDC plus IL-2, expression of KIR on CD56^dim^CD16^high^ NK cells was significantly diminished (Figure 2D).

In contrast to KIRs, the NKG2A/CD94 receptor displays its highest expression on CD56^bright^CD16^neg^ cells and is reduced during the presumptive maturation to CD56^dim^CD16^high^ cells [28] (Figure 2E and Suppl. Fig. 2). When we evaluated NKG2A/CD94 expression in untreated AML patients, no significant differences of expression levels compared to healthy individuals were found for CD56^dim^CD16^high^ NK cells. However, the percentage of cells expressing NKG2A/CD94 was significantly reduced in the CD56^bright^CD16^neg^ subset. Following remission-induction chemotherapy, both CD56^bright^ subsets showed a significant increase in expression of NKG2A/CD94. HDC plus IL-2 treatment further increased this expression even beyond levels observed in healthy individuals (Figure 2E).

### IL-2 is required for normal expression of NKG2D, NKp30, and NKp46 and increases NKG2A/CD94 expression on NK cells

**Figure 3:**
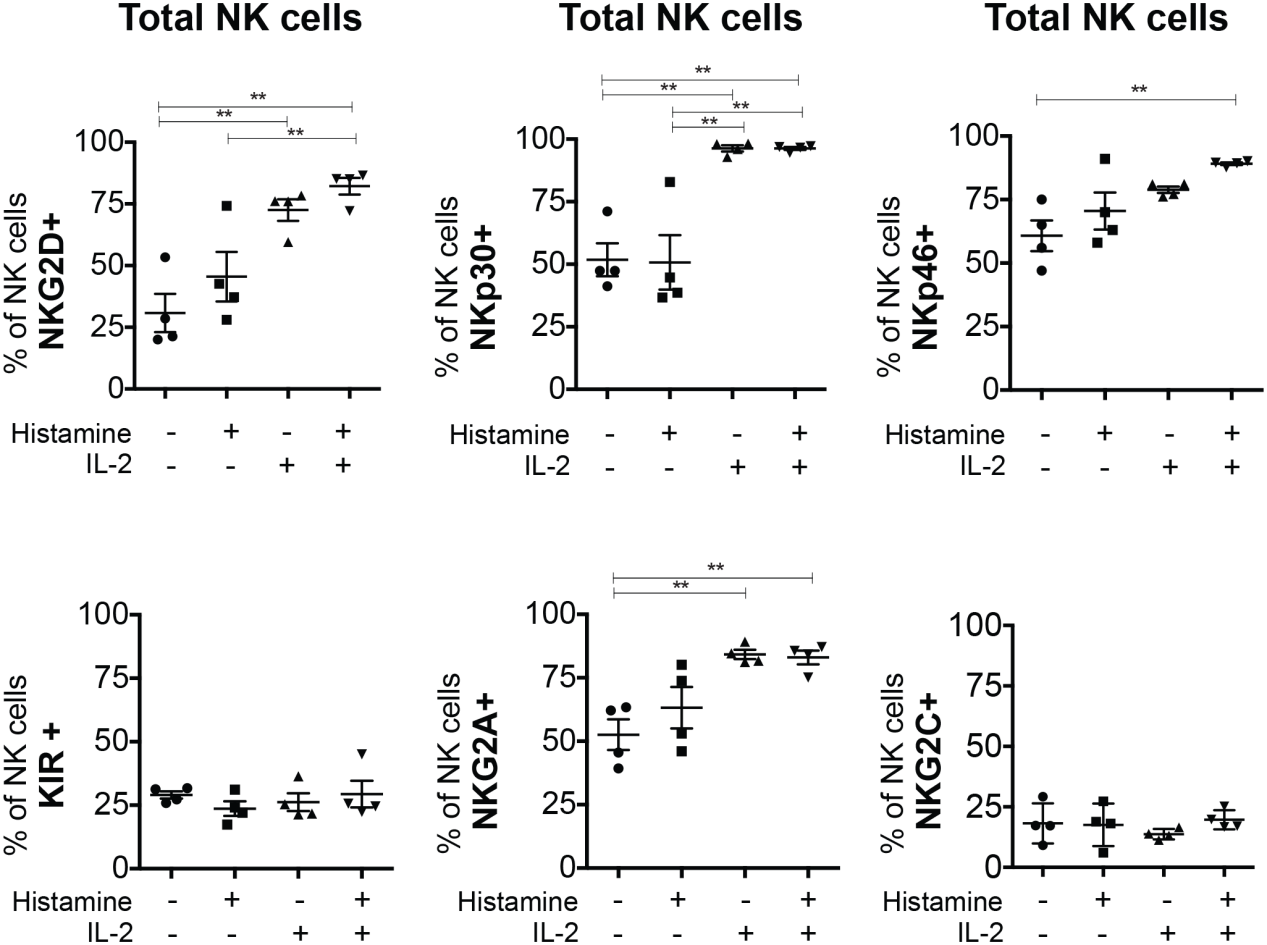
*In vitro* effect of HDC and IL-2 on the expression of NK cell markers in total NK cells PBMC from 4 healthy donors were cultured without or with HDC (10^−5^ M), IL-2 (500 UI/ml) or both compounds for 6 days. NK cells expressing the receptors NKG2D, NKp30, NKp46, KIR, NKG2A/CD94 and NKG2C/CD94 were determined as the number of positively stained cells minus the number of cells stained with an isotype-matched negative control antibody. Mean values +/− SD of the percentages of positive cells within the CD56^pos^CD3^neg^ NK cell fraction are shown. *p<0.05, **p<0.01

To determine to what extent IL-2 or HDC affect receptor expression, we studied NK cells cultured *in vitro* in the presence or absence of the compounds for 6 days. We noticed that IL-2 was required to maintain high levels of NKG2D, NKp30 and NKp46 expression on cultured NK cells. HDC alone appeared to have only a minimal effect on NKG2D and NKp46 expression and in combination with IL-2 could slightly further increase the expression of these receptors. These small effects of HDC on NKG2D and NKp46 expression were seen in the total fraction of NK cells (Figure 3) and also in the individual subsets examined (Suppl. Fig. 3).

The expression of KIR and NKG2C was unaffected by HDC and IL-2 treatment *in vitro* (Figure 3 and Supp. Figure 3D). IL-2 but not HDC increased the expression of NKG2A/CD94 on total NK cells (Figure 3) and the effect is visible on all NK cell subsets (Suppl. Figure 3E).

### The CD56^bright^CD16^neg^ and CD56^bright^CD16^low^ subpopulations specifically expand in response to IL-2 *in vitro*

**Figure 4:**
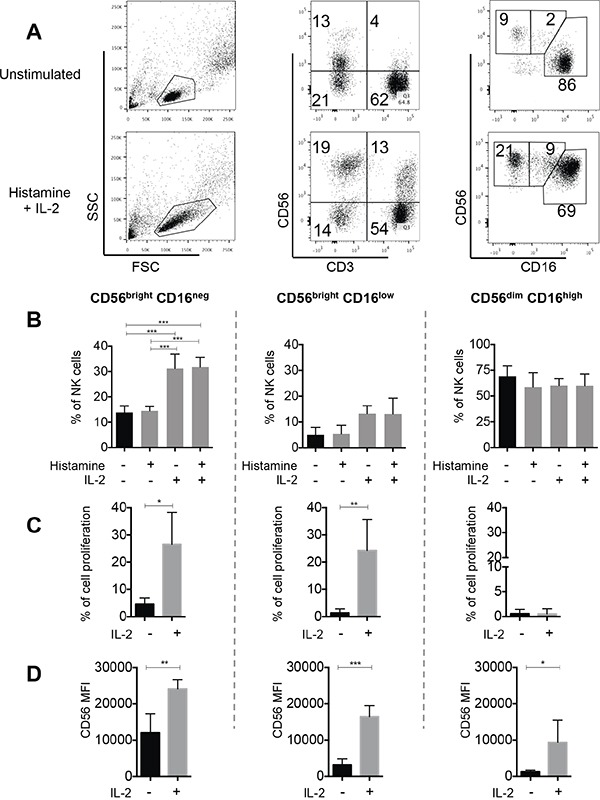
*In vitro* effect of HDC and IL-2 on NK cell proliferation To study expansion of NK cell subsets *in vitro*, eFluor670-labeled total PBMC from 5 healthy donors were cultured without or with addition of HDC (10^−5^ M), IL-2 (500 UI/ml) or HDC plus IL-2 for 6 days. NK cells were then identified as CD56^pos^CD3^neg^ and the NK cell subsets as CD56^bright^CD16^neg^, CD56^bright^CD16^low^ or CD56^dim^CD16^high^ cells. **A.** Representative dot plots are shown for one individual. **B.** The mean percentages of all three NK cell subsets within the total NK cell fraction were calculated from 5 individuals. **C.** To directly study the proliferation of single subsets, NK cells were isolated from eFluor670-labeled PBMC by negative NK cell selection followed by cell sorting. Cell proliferation of the isolated subsets was determined after 6 days of culture without or with IL-2 by dye dilution analysis. Cell proliferation is displayed as the percentages of cells with reduced intensities of eFLuor-670 labeling in comparison to peak values at day 0. **D.** In addition, the intensities of cell surface expression of CD56 was analyzed in the single cultured NK cell subsets Mean values +/− SD are shown for B-D. *p<0.05, **p<0.01, ***p<0.001

The remarkable increase in both CD56^bright^ subsets after HDC plus IL-2 treatment, led us to further investigate this *in vitro*. HDC plus IL-2 caused a significant expansion of the CD56^bright^CD16^neg^ and CD56^bright^CD16^low^ NK cell subpopulations after culturing *in vitro*, which partly mimicked the *in vivo* effect in patients. IL-2 alone caused a comparable effect, whereas HDC by itself did not induce any detectable changes (Figure 4A, 4B). Next we evaluated proliferation of the subsets during culturing *in vitro*. The CD56^bright^CD16^neg^ subset of NK cells showed high proliferative potential, whereas the more mature CD56^dim^CD16^high^ subpopulation did not divide. It was intriguing that the CD56^bright^CD16^low^ cell subset displayed also an outstanding proliferative capability under IL-2 stimulation, similar to that of CD56^bright^CD16^neg^ cells (Figure 4C). Furthermore, IL-2 stimulation was associated with an increased intensity of expression of CD56 on all three NK cell subsets (Figure 4D). The differential expression of CD56 between the different subsets was maintained, with the highest intensities displayed in CD56^bright^ and gradual decreased intensities over CD56^bright^CD16^low^ to CD56^dim^ cells (Figure 4D).

### The CD56^bright^CD16^neg^ and CD56^bright^CD16^low^ subsets display similar IL-2 dependent capacity to degranulate and produce cytokines

We were further interested to determine the potential of the NK cell subsets to degranulate and to produce cytokines after *in vitro* culture of a complete PBMC fraction in the presence or absence of IL-2. Our group and others [29] have seen that CD16 is downregulated after culture in medium alone or after cytokine stimulation; thus, a correct identification of the single NK cell subsets based on CD16 expression is hampered when cultured over five days. We therefore designed a polychromatic method based on cell-tracing to be able to follow up all three subsets over a five-day incubation period regardless of CD16 expression. In short, we separated the three NK cells subsets by FACS sorting and labeled them subsequently with different cell trackers. The differentially labeled subsets of the same donor were then recombined and cultured together with an unstained PBMC fraction from the same donor to mimic a physiological combination of cells (see Materials and Methods and Suppl. Fig. 4). After five days of *in vitro* culture, CD107a expression and cytokine production in response to U937 cells was determined for the different subsets identified by their individual cell trackers in flow cytometry.

**Figure 5:**
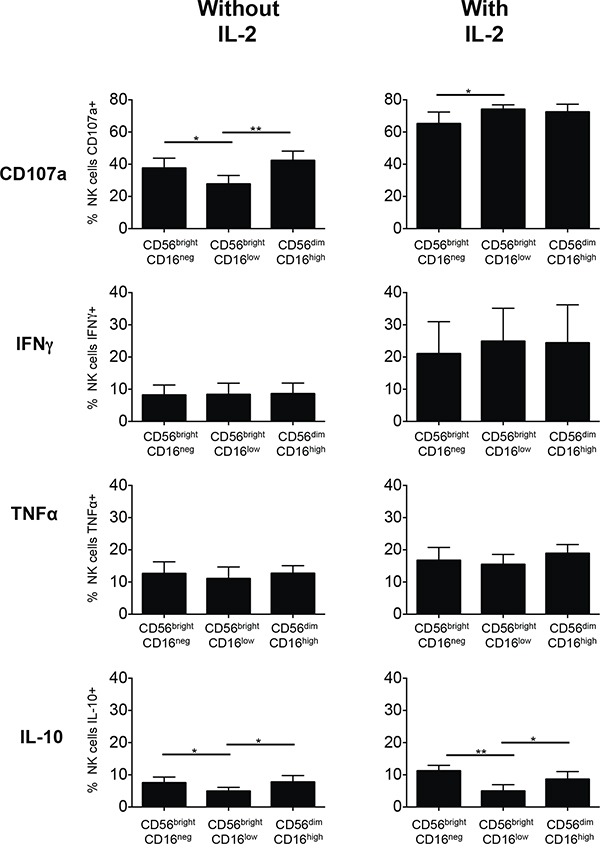
Degranulation and cytokine production of subsets of NK cells from healthy donors cultured without or with IL-2 Individual NK cell subsets were isolated from PBMCs of healthy donors by negative NK cell selection followed by preparative cell sorting and labeled with either CFSE (CD56^bright^CD16^neg^), eFluor670 (CD56^bright^CD16^low^) or CellTrace Violet (CD56^dim^CD16^high^). All three individually labeled NK cell subsets were then again combined and a complete PBMC culture reconstituted as described in Materials and Methods and depicted in Suppl. Fig. 4. The cells were cultured for 5 days without or with addition of IL-2. Then the cells were stimulated by addition of the leukemic target cell line U937 in a 5:1 effector:target ratio for 6 h. The percentages of cells displaying CD107a within each NK cell subset were determined. Cells producing cytokines were detected after intracellular cytokine staining and displayed as percentages of cells positive for IFN-γ, TNF-α or IL-10 within each NK cell subset. Mean values of the percentages of stained cells +/− SD were calculated from the results obtained for 5 healthy donors and are shown for the CD56^bright^CD16^neg^, CD56^bright^CD16^low^ and CD56^dim^CD16^high^ NK cells subsets. *p<0.05, **p<0.01

Following *in vitro* culture with IL-2 all three subsets displayed higher capacities to degranulate and to produce IFN-γ when compared to cultures without IL-2 (Figure 5). This difference was less pronounced for TNF-α and IL-10 was produced only by a small proportion of the subsets. Generally the capacities to degranulate and to produce cytokines were similar for all three subsets, although degranulation activity of CD56^bright^CD16^low^ cells seemed to be lower in the absence of IL-2 and this subset appeared also to produce less IL-10.

### A diminished capacity to produce IFN-γ is found to be increased after chemotherapy and maintained at high level during HDC plus IL-2 treatment in AML patients

**Figure 6:**
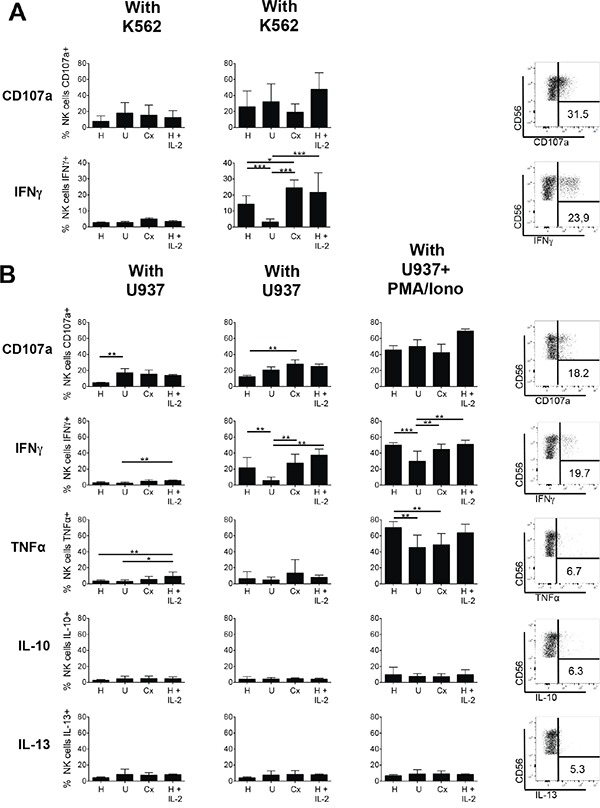
Degranulation and cytokine production of NK cells of healthy donors and AML patients PBMC from healthy donors (H), untreated AML patients (U), patients after chemotherapy (Cx) and patients treated with HDC plus IL-2 for 3 weeks were analyzed. **A.** Degranulation and IFN-γ production of CD56^+^CD3^−^ NK cells without or with stimulation by the target cell line K562: Total PBMCs were cultured without or with K562 in a 5:1 effector:target ratio for 6 h. Then degranulation was measured as the percentage of NK cells displaying CD107a on the surface. IFN-γ production was determined after intracellular cytokine staining as percentage of NK cells displaying staining for IFN-γ. Mean values +/− SD are shown. (H: n=18; U: n=11; Cx: n=6; HDC+IL2: n=8). *p<0.05. Exemplary dot plots for CD107a and IFN-γ staining after stimulation with K562 cells are depicted for one exemplary patient treated with HDC plus IL-2 (right panels). **B.** Degranulation and cytokine production of CD56^+^CD3^−^NK cells without or with stimulation by the target cell line U937 or additional stimulation with PMA plus ionophore: Total PBMCs were cultured without or with U937 in a 5:1 effector:target ratio for 6 h. Where indicated in addition to U937 cells PMA plus ionophore was added to the cultures to achieve a full response. CD107a surface expression and intracellular staining for IFN-γ, TNF-α, IL-10 and IL-13 were assessed using the respective antibodies as described above. Shown are mean values +/− SD. (H: n=14; U: n=7; Cx: n=6; HDC plus IL-2: n=3). * p<0.05. Exemplary dot plots of staining for CD107a and the cytokines IFN-γ, TNF-α, IL-10 and IL-13 following stimulation with K562 or U937 cells are depicted for one patient treated with HDC plus IL-2 (right panels).

To determine the effect of chemotherapy followed by HDC plus IL-2 on the functional capabilities of NK cells in AML patients, we analyzed first the intracellular production of IFN-γ and of the marker CD107a that typically increases on the cell surface during degranulation. In the absence of K562 target cells, NK cells of healthy individuals, of untreated AML patients, of patients after chemotherapy and of HDC plus IL-2-treated patients displayed only low spontaneous capabilities to produce IFN-γ or to display CD107a (Figure 6A, left panels). Following stimulation with K562 target cells, NK cells from healthy individuals displayed increased production of IFN-γ, however in untreated AML patients K562-induced IFN-γ production was strongly impaired. Following chemotherapy, the capacity, to produce IFN-γ was increased and maintained at high level during HDC plus IL-2 treatment (Figure 6A, lower right panel). The capacity to upregulate the degranulation-related surface antigen CD107a in response to K562-cell mediated activation did not show similar significant differences between healthy individuals, untreated AML patients and patients after chemotherapy, but seemed to be somewhat increased after HDC plus IL-2 treatment (Figure 6A, upper right panel).

We then further evaluated the reactivity of NK cells towards the leukemic cell line U937 in a second series. Again, the capacity to upregulate CD107a and to produce IFN-γ as well as the additional cytokines TNF-α, IL-10 and IL13 was determined (Figure 6B). In accordance with the results obtained with K562 cells, the capacity to produce IFN-γ was strongly diminished in untreated AML patients, normalized in patients after chemotherapy, and maintained or slightly further increased in HDC plus IL-2 treated patients. This pattern was also visible when, as a control, PMA plus ionophore were added in addition to the U937 cells to achieve a full response, although under these conditions NK cells from untreated patients were also significantly activated for IFN-γ production. With regard to TNF-α, IL-10 and IL13 only very low production levels with no clear differences were obtained for all samples and U937 stimulation. Following the additional stimulation with PMA plus ionophore high production of TNF-α became visible, but IL-10 and IL-13 remained low. In contrast to IFN-γ production, the capacity of NK cells to upregulate CD107a in response to U937 cell-induced activation was not reduced in untreated AML patients, but was rather somewhat increased in patients after chemotherapy when compared to healthy controls. No additional increase after HDC plus IL-2 treatment was visible.

## DISCUSSION

Several reports have described that NK cells are frequently present in low numbers in peripheral blood of AML patients and display impaired functions, partially due to a low expression of activating receptors such as NCRs [3, 4, 30, 31]. In line with these findings we have observed diminished expression of the activating NK receptors NKp30, NKp46 and NKG2D as well as a lower capacity of NK cells to produce IFN-γ in untreated AML patients at the time of diagnosis. Taken together with results of hematopoietic SCT in AML, which have shown that rapid recovery of NK cells after transplantation is an indicator of improved clinical outcome [12], it appears that NK cells are of considerable importance for the control of AML, and restoring the NK cell fraction should be an important goal in the overall treatment plan. We show in this context that chemotherapy achieves a significant increase in activating receptors and in the capacity of NK cells to produce IFN-γ, however the size of the NK cell fraction remains diminished.

Furthermore, maintenance therapy with HDC plus IL-2 after chemotherapy in AML patients has been proposed with the hope to restore a diminished NK cell system. In this regard, a clinical phase II trial of maintenance therapy with HDC plus IL-2 after chemotherapy in AML patients has shown reduced relapse rates [18], however the effects of the treatment on NK cells have not been investigated in detail in this study.

We assessed here the effect of HDC plus IL-2 on the total NK cell fraction and on NK cell subsets. An outstanding three-fold expansion of CD56^bright^ NK cells driven by treatment with HDC plus IL-2 in AML patients after chemotherapy was observed. In distinction to NK cells from healthy individuals, two clear subsets of CD56^bright^ NK cells each comprising 20 to 30 % of the total NK cells could be distinguished. These two CD56^bright^ populations were characterized by the absence or low presence of CD16, a marker used to identify the cytotoxic and rather mature CD56^dim^ subset. This suggests differences in function and/or developmental stages between the CD56^bright^CD16^neg^ and CD56^bright^CD16^low^ subsets.

In support of this possibility, the CD56^bright^CD16^low^ subset showed intermediate expression levels of the markers CD57, KIR and NKG2A/CD94 between the levels seen in CD56^bright^CD16^neg^ and CD56^dim^CD16^high^ NK cells, suggesting that this population is in progress to acquire features of the more mature CD56^dim^ subset [28]. This is in line with the suggestion of Béziat et al that CD56^bright^CD16^+^ NK cells are in a functional differentiation stage between CD56^bright^ and CD56^dim^ NK cells [32]. Although it can currently not be excluded that CD56^bright^ and CD56^dim^ cells could also be separate lineages, a likely precursor relationship of the more immature CD56^bright^ to the more mature CD56^dim^ cells has been widely proposed as discussed recently in detail by Michel et al. [26].

Generally, the CD56^bright^ cells are considered the major cytokine producers, whereas the CD56^dim^ population is the more cytotoxic subset being less efficient in producing IFN-γ in response to specific cell activation [33]. However, the differential cytokine secretion described for the CD56^bright^ and CD56^dim^ remains controversial [34] and it has been described by others that the CD56^dim^ population can become a rapid and competent IFN-γ producer upon activation [35] and recognition of target cells [36, 37].

The occurrence of high proportions of CD56^bright^ NK cells in peripheral blood has been observed previously, for example after hematopoietic stem cell transplantation [38]. In this case it was proposed that these cells are activated mature NK cells that expand in patients with low numbers of T cells. The expansion did not correlate with new hematopoiesis and the CD56^bright^ NK cells prevailed for prolonged periods. In a different approach the effect of IL-2 on purified clinical scale donor NK cells was followed over 14 days in culture and variable donor-dependent expansion of CD56+CD16- observed [39], the cells displayed increased activating receptor expression and cytokine production.

When we cultured NK cells from healthy individuals *in vitro* with HDC plus IL-2, the expansion of a CD56^bright^CD16^neg^ and a CD56^bright^CD16^low^ subset observed after HDC plus IL-2 treatment in AML patients could be partly mimicked. Moreover, in a proliferation assay with isolated subsets we could show that this is primarily due to a direct effect of IL-2 on the CD56^bright^ subsets. IL-2 specifically triggered proliferation of both CD56^bright^ subsets, whereas CD56^dim^ cells did not proliferate. HDC by itself had no detectable effects on proliferation *in vitro*.

This selective high proliferative potential of the CD56^bright^CD16^neg^ and CD56^bright^CD16^low^ cells in response to IL-2 is in line with the possibility that HDC plus IL-2 therapy triggers proliferation of the small subpopulation of more immature CD56^bright^CD16^neg^ cells present in AML patients, leading to an expansion and parallel differentiation first to CD56^bright^CD16^low^ cells, which further proliferate, and then, at least in part, mature to CD56^dim^CD16^high^ NK cells.

This selective CD56^bright^ expansion might be further supported by the fact that CD56^bright^ cells are more resistant to reactive oxygen species and possess a higher anti-oxidative capacity than the CD56^dim^ subset [40].

Considering that the estimated half-life of NK cells in peripheral blood is about 2 weeks and that they can expand only within a certain limit [41], one would expect that after two to three weeks of HDC plus IL-2 therapy a major portion of the NK cell pool would have been reconstituted and expanded under therapy conditions.

Whereas monotherapy with IL-2 has not shown efficacy in AML, treatment with HDC plus IL-2 reduces relapse in patients with AML [18]. Available *in vitro* data support that histamine protects NK cells and other lymphocytes from phagocyte-mediated functional inhibition by suppression of ROS formation [42]. Furthermore, it was reported that it can potentiate the effect of IL-2 on the cytotoxic capability of NK cells towards human leukemic blasts [43]. Principally, NK cells possess the histamine receptor H4R [44] and histamine could have also direct effects on proliferation and receptor expression of NK cells. However, our *in vitro* data show that histamine alone does not exert substantial effects on the expansion of the CD56^bright^ subsets and could not potentiate the effect of IL-2 on this expansion. It is possible that it rather contributes to survival *in vivo* and improved chemotaxis [44] and not to proliferation of NK cells.

The results on receptor expression on NK cells of AML patients after chemotherapy and after treatment with HDC plus IL-2 together with the *in vitro* data on receptor expression highlight the effects of the chemotherapy to regain expression of the activating receptors NKG2D, NKp30 and NKp46 on NK cells of patients and support the function of IL-2 to maintain high expression. *In vitro*, IL-2 was necessary in the culture medium to maintain normal high expression levels of these activating receptors over several days. This holds true for all three NK cell subsets tested, the CD56^bright^CD16^neg^, CD56^bright^CD16^low^ and CD56^dim^CD16^high^. This suggests that IL-2 is necessary for appropriate expression of the activating receptors on all NK cells. In the case of NKG2D and NKp46 HDC displayed a small effect by itself and could somewhat increase the effects of IL-2. This is in line with previous findings that exposure to histamine preserves the expression of these receptors [45]. However, the relatively small effects of HDC on receptor expression suggests that the major contribution of HDC may rather be in the inhibition of ROS and thus protection of lymphoid cells from inactivation [45].

KIR are usually not or only minimally expressed on the less mature CD56^bright^CD16^neg^ cells, but are rather acquired late during NK cell maturation leading to high cell numbers with KIR expression only in the CD56^dim^CD16^high^ subset [27]. In AML patients treated with HDC plus IL-2, CD56^dim^CD16^high^ NK cells displayed reduced expression of KIR, suggesting that the majority of these cells had just been differentiated from CD56^bright^ cells and may not have yet acquired full KIR expression levels.

In contrast to the KIR, the NKG2A/CD94 receptor displays highest expression on CD56^bright^CD16^neg^ cells and is reduced during the presumptive maturation to CD56^dim^CD16^high^ cells [28]. In newly diagnosed untreated AML patients, the NKG2A/CD94 receptor was expressed on a lower number of the CD56^bright^ cells than in healthy individuals. After chemotherapy the levels of expression in the CD56^bright^CD16^neg^ subset were normalized and upon treatment with HDC plus IL-2, a further increased of levels beyond those seen in healthy donors was observed. Even in the CD56^dim^CD16^high^ subset an unusually high number of cells expressed NKG2A. As discussed in the context of KIR this indicates a less mature phenotype of CD56^dim^CD16^high^ NK cells generated to a large extent during treatment, which may further mature over time.

We have further analyzed the capacity of NK cells from patients to upregulate CD107a expression (as indirect marker of degranulation) and to produce several cytokines in response to the standard target cell line K562 or the leukemic cell line U937. We did not observe a significant deficiency of degranulation for the NK cells of untreated patients. However, we detected, in newly diagnosed untreated patients, a significant major defect in the capacity of NK cells to produce IFN-γ in response to K562 and U937 cells. This was found normalized in patients after chemotherapy and during additional treatment with HDC plus IL-2 the high capacities to produce IFN-γ were maintained and partly further supported. This was in part mimicked at a lower level by another immune stimulatory cytokine, TNF-α. Two other cytokines involved in the negative control of immune activation, IL-10 and IL-13, were expressed only in a small fraction of NK cells irrespective of the treatment. This supports that secretion of immune-inhibitory cytokines is not altered in AML or modulated during therapy.

After this work has been submitted, two additional reports from the Hellstrand group independently described an expansion of CD56^bright^CD16^neg^ cells in HDC plus IL-2 treated AML patients [46, 47], but the CD56^bright^CD16^low^ cells were not separately investigated in these studies. Furthermore, somewhat higher intensities of expression of the NCR NKp30 and NKp46 were observed after 3 weeks of treatment and the data supported that a high expression of NKp30 and NKp46 before and during therapy in older patients is a predictor of leukemia-free and overall survival.

Taken together, we propose that the improved leukemia-free survival in AML patients treated with HDC plus IL-2 might, at least to some extent, result from the induced expansion of CD56^bright^ NK cells reconstituting a fresh pool of immunocompetent NK cells including the three described NK cell subsets and additionally supporting and maintaining a high expression level of activating receptors and a high capacity of IFN-γ production and degranulation capability.

## MATERIALS AND METHODS

### Patients and healthy donors

A complete list of blood donors comprising untreated AML patients at time of diagnosis, patients after chemotherapy, patients additionally treated with HDC plus IL-2 and healthy individuals is provided as Suppl. Table 1. Patients received induction chemotherapy with daunorubicin (45 mg/m^2^ i.v. days 1-3), cytosine arabinoside, ARA-C (2x 100 mg/m^2^ i.v. days 1-7) and etoposide (100 mg/m^2^ i.v., days 1-5) and 3 or 4 cycles of consolidation chemotherapy with high or intermediate dose ARA-C (i.e. 2x 3g/m^2^, days 1,3,5 or 2x 1g/m^2^, days 1,3,5, respectively) [48–50]. After achieving hematologic remission, patients were treated with HDC (0.5 mg) plus recombinant IL-2 (16400 IU/Kg) twice-daily s.c. for 21 days per cycle, according to an established protocol [18]. The study was approved by the local ethics committee of the Medical University of Vienna (protocol number EK 1667/2014) and all patients and donors gave written informed consent to participate.

### Multicolor flow cytometry

Peripheral blood mononuclear cells (PBMC) were isolated from 9 ml EDTA tubes by gradient centrifugation in cell separation media (Lympholyte®-H, Cedarlane Labs, Burlington, Ontario). Cells were then stained with fluorochrome-conjugated monoclonal antibodies (mAb) (Supp. Table 2) using a 6 to 8-color immunophenotyping panel (Supp. Table 3) and analyzed by flow cytometry. Discrimination of live and dead cells was performed according to the manufacturer´s protocols (LIVE/DEAD® Fixable Aqua Dead Cell Staining Kit; Life Technologies, Oregon, USA). Total NK cells were identified as CD56^pos^CD3^neg^ cells and NK cell subsets as CD56^bright^CD16^neg^, CD56^bright^CD16^low^ and CD56^dim^CD16^high^. Positive cells for a specific marker were determined as the percentage of positively stained cells minus the number of cells stained with an isotype-matched negative control antibody. Acquisition was performed either on a FACS Canto II or LSRII (BD Biosciences, San Jose, CA, USA). Flow cytometry analyses were performed with FACSDiva software v6.0 (BD Biosciences) and FlowJo vX (Tree Star, San Carlos, CA, USA).

### *In vitro* cultures of NK cells to study receptor expression under influence of histamine and IL-2

PBMC were cultured in RPMI 1640 medium (Gibco; Paisley, UK), supplemented with 10% FCS (Biochrom; Berlin, Germany), 10 mM HEPES (Sigma), 1 mM L-Glutamine (Gibco), 100 U/ml penicillin and 100 μg/ml streptomycin (both from PAA; Pasching, Austria), containing either 10^−5^ M HDC (Sigma-Aldrich; Steinheim, Germany) or 500 IU IL-2 (Proleukin, Novartis) or the combination of HDC plus IL-2 for 6 days. Then receptor expression was determined by antibody staining and flow cytometry as described above.

### Cell proliferation and measurement of CD56 intensities

PBMC were labelled with 5μM eFluor670 (eBioscience, San Diego, CA, USA) and cultured for 5 days. Then proliferation was determined by the loss of dye-labeling in comparison to peak levels at day 0. In the same cultures, staining with CD56 mAb was performed and the expression level of CD56 assessed by the median fluorescence intensity (MFI).

### Cell tracing method for individual NK cell subsets *in vitro*

NK cells were negatively selected from PBMCs using the MACS NK cell isolation kit (Miltenyi Biotec, Bergisch Gladbach, Germany) and the three NK cell subsets (CD56^bright^CD16^neg^, CD56^bright^CD16^low^ and CD56^dim^CD16^high^) isolated following staining with anti-CD56-PE and anti-CD16-FITC and preparative cell sorting on a FACSAria. The three subsets were then individually labelled with either 2 μM CFSE (Biolegend), 5μM eFluor670 (eBioscience, San Diego, CA, USA) or 5μM CellTrace Violet (Life Technologies) (see also Suppl. Fig. 4). The differentially labeled subsets were then again combined and a complete PBMC fraction reconstituted by adding back the stored NK cell-depleted fraction obtained during negative NK cell selection. This reconstituted PBMC fraction containing all three subsets individually labelled was then cultured and analyzed for degranulation and cytokine production as described below.

### CD107a expression and cytokine production in NK cells

A total of 10^6^ PBMC or remixed PBMC fraction (containing 1×10^5^ recombined dye-labeled NK cells prepared as described under cell tracing method) was co-cultured with K562 or U937 target cells (obtained from ATCC) in 500 μl cell culture medium for 6 hours at 37°C using an optimized effector:target ratio of 5:1. For CD107a expression, the CD107a mAb was added to the cells at the beginning of the culture. In order to block degradation of reinternalized CD107a and cytokine secretion, respectively, 2 μ molar monensin and 10 μg/ml of brefeldin A (GolgiStop and GolgiPlug, BD Biosciences) were added to the culture 1 h after start of the co-culture. In certain cases 50 ng/ml phorbol 12-myristate 13-acetate (PMA, Merck, Darmstadt, Germany) and 1 μg/ml ionomycin (Cayman Chemical Co., Ann Harbor, MI) were added in addition to achieve a full activation. After incubation for additional 5 h, cells were then first stained on the surface with anti-CD56 and -CD3 antibodies. Then fixation and permeabilization of the cells were performed using Cytofix/Cytoperm solution (BD Biosciences). To assess cytokine production, the fixed and permeabilized cells were further stained with antibodies for IFN-γ, TNF-α, IL-10 and IL13 (Suppl. Table 2). Then flow cytometry was performed. As controls, PBMC were cultured without target cells to display their spontaneous CD107a expression and cytokine production.

### Statistical analysis

Prism 6 software (GraphPad, San Diego, CA) was used for statistical analysis. For multiple comparisons within a data group, an ordinary one-way ANOVA was performed followed by Bonferroni´s Multiple Comparison Test.

## SUPPLEMENTARY TABLES AND FIGURES




