# Supplementary material for: Maintenance therapy with histamine plus IL-2 induces a striking expansion of two CD56bright NK cell subpopulations in patients with acute myeloid leukemia and supports their activation
Source: Oncotarget. 2016 Jun 21;7(29):46466–81. doi: 10.18632/oncotarget.10191 (PMC5216810; doi:10.18632/oncotarget.10191)
Supplement: Supplementary file 1 [file oncotarget-07-46466-s001.pdf]

Maintenance therapy with histamine plus IL-2 induces a striking expansion of two CD56^{bright} NK cell subpopulations in patients with acute myeloid leukemia and supports their activation

SUPPLEMENTARY TABLES AND FIGURES

Supplementary Table S1: List of AML patients and control healthy donors. Compilation of the 11 untreated AML patients (average 62 years, range 23-83 years), 9 AML patients after chemotherapy (average 49 years, range 28-72 years), 9 AML patients treated in addition with HDC plus IL-2 (average 47, range 21-69 years) and the 48 healthy donors (average 43 years, range 23-83 years) from whom blood samples were obtained.

See Supplementary File 1

Supplementary Table S2: List of antibodies used for identification of NK cells by flow cytometry

mAb anti-	Conjugated Fluorochrome	Clone	Company
CD107a	FITC	H4A3	BD Pharmingen
CD16	APC-Cy7	3G8	BD Pharmingen
CD20	PE	2H7	Biolegend
CD3	PerCP-Cy5.5	UCTH1	BD Pharmingen
CD3	V450	SK7	BD Biosciences
CD45	V500	HI30	BD Biosciences
CD56	PE-Cy7	B159	BD Pharmingen
CD57	APC	NK-1	BD Pharmingen
CD94	FITC	HP-3D9	BD Pharmingen
IFN-γ	APC-Cy7	4S.B3	Biolegend
IgG1	APC	IS5-21F5	Miltenyi Biotec
IgG1	FITC	IS5-21F5	Miltenyi Biotec
IgG1	PE	IS5-21F5	Miltenyi Biotec
IL-10	PE	JES3-19F1	BD Pharmingen
IL-13	PerCP-Cy5.5	JES10-5A2	Biolegend
NKG2A	APC	Z199	BD Pharmingen
NKG2C	PE	134591	R&D Systems
NKG2D	APC	1D11	BD Pharmingen
NKp30	APC	p30-15	BD Pharmingen
NKp46	APC	9E2/NKp46	BD Pharmingen
pan-KIR	FITC	180704	R&D Systems
TNFα	PECY7	MAb111	Biolegend

Supplementary Table S3: Combination of markers used for flow cytometry

Cell surface staining					Intracellular staining	
**1**	**2**	**3**	**4**	**5**	**6**	**7**
CD56	CD56	CD56	CD56	CD56	CD56	CD56
CD3	CD3	CD3	CD3	CD3	CD3	CD3
CD16	CD16	CD16	CD16	CD16	CD45	CD16
CD45	CD94	KIR	CD158a	NKG2D	IFN-γ	IFN-γ
CD14	NKG2A	NKP30	CD57	NKP46	TNF	CD107a
CD20	NKG2C	NKP44	CD158b		IL-10<br>IL-13	eFluor-670
Live/dead	Live/dead	Live/dead	Live/dead	Live/dead	CD107	Live/dead
					Live/dead	

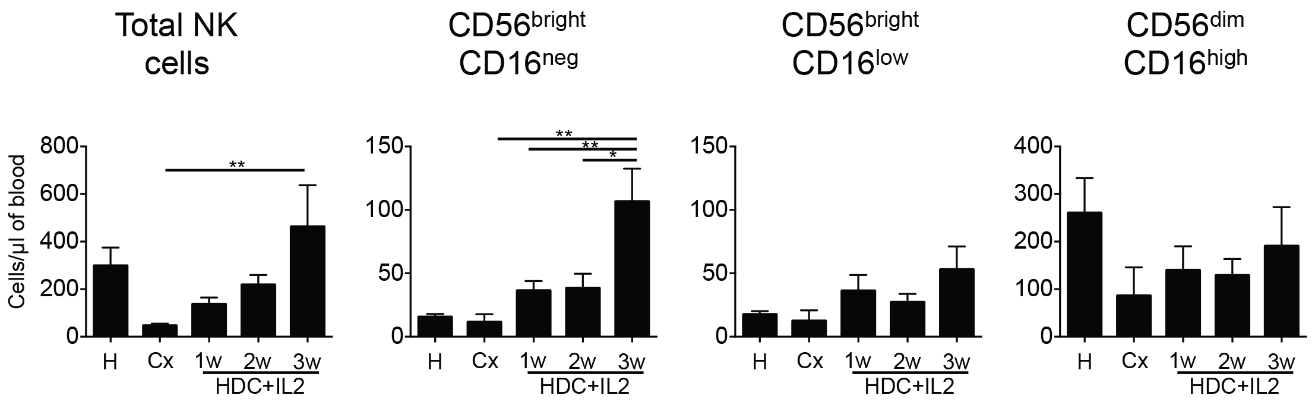

Supplementary Figure S1: Absolute numbers of NK cells in AML patients and healthy donors. Absolute cell numbers of total NK cells and of single NK cell subsets from 12 healthy donors (H), 9 patients after chemotherapy (Cx) and patients undergoing additional HDC plus IL-2 therapy after 1, 2 and 3 weeks (w) of treatment with HDC plus IL-2 (1w: n=9; 2w: n=5; 3w: n=6) are shown. The calculation is based on the total leukocyte count and the percentages obtained by flow cytometry for the proportion of $CD3^{neg}CD56^{pos}$ cells (total NK cells) and the $CD56^{bright}CD16^{neg}$, $CD56^{bright}CD16^{low}$ and $CD56^{dim}CD16^{high}$ cells. Mean values +/- SD are shown. *$p<0.05$, **$p<0.01$.

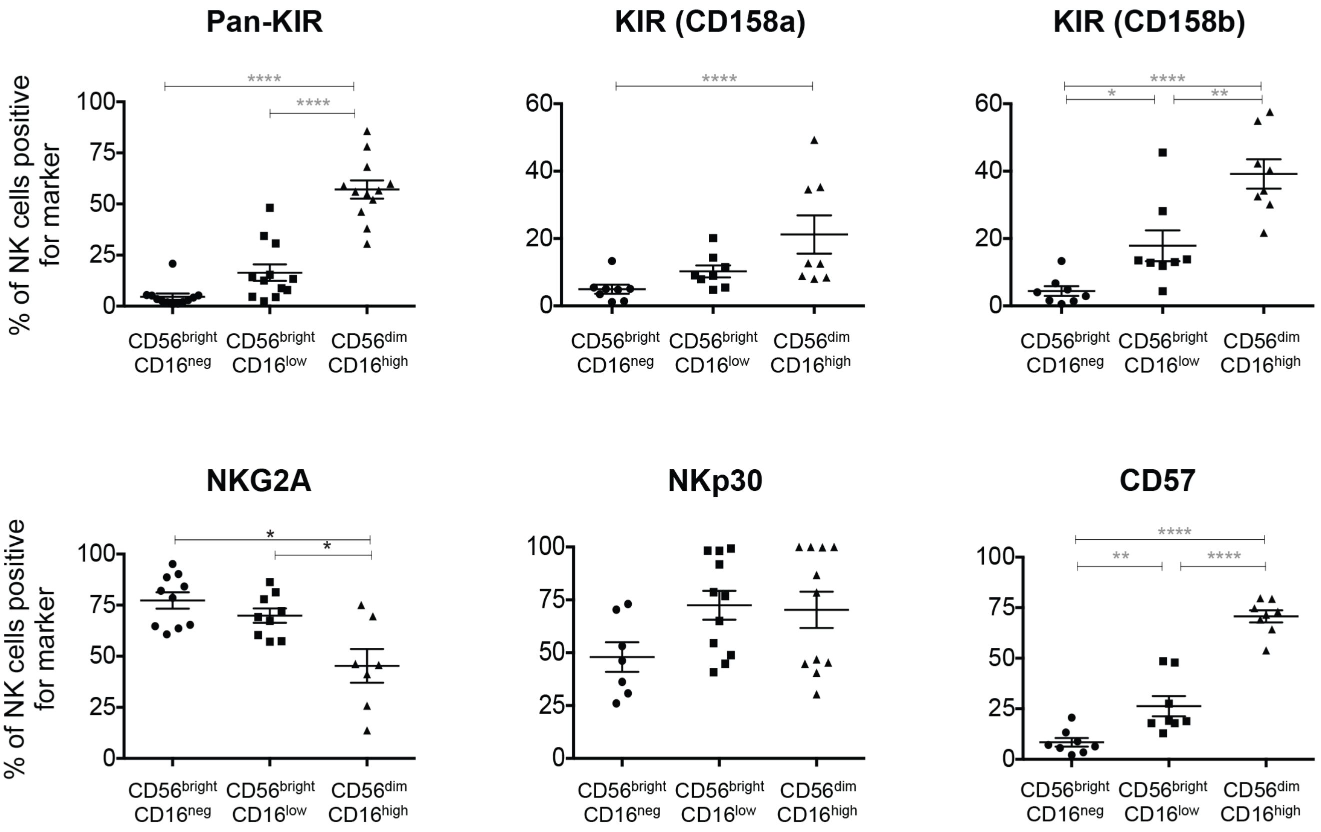

Supplementary Figure S2: Expression patterns of cell markers in NK cell subsets of healthy donors. PBMC from 9 healthy donors were stained with anti-CD3, -CD56 and -CD16 to identify the $CD56^{bright}CD16^{neg}$, $CD56^{bright}CD16^{low}$ and $CD56^{dim}CD16^{high}$ cell populations. Cells were further stained with anti-pan-KIR, -CD158a, -CD158b, -NKG2A, -NKp30 or -CD57. The expression pattern of the markers in the different NK cell subsets were determined by flow cytometry. Shown are the percentages of NK cells positive for the corresponding marker within the individual subsets. Median and SEM are shown. *p<0.05, **p<0.01, ***p<0.001, **** p<0.0001.

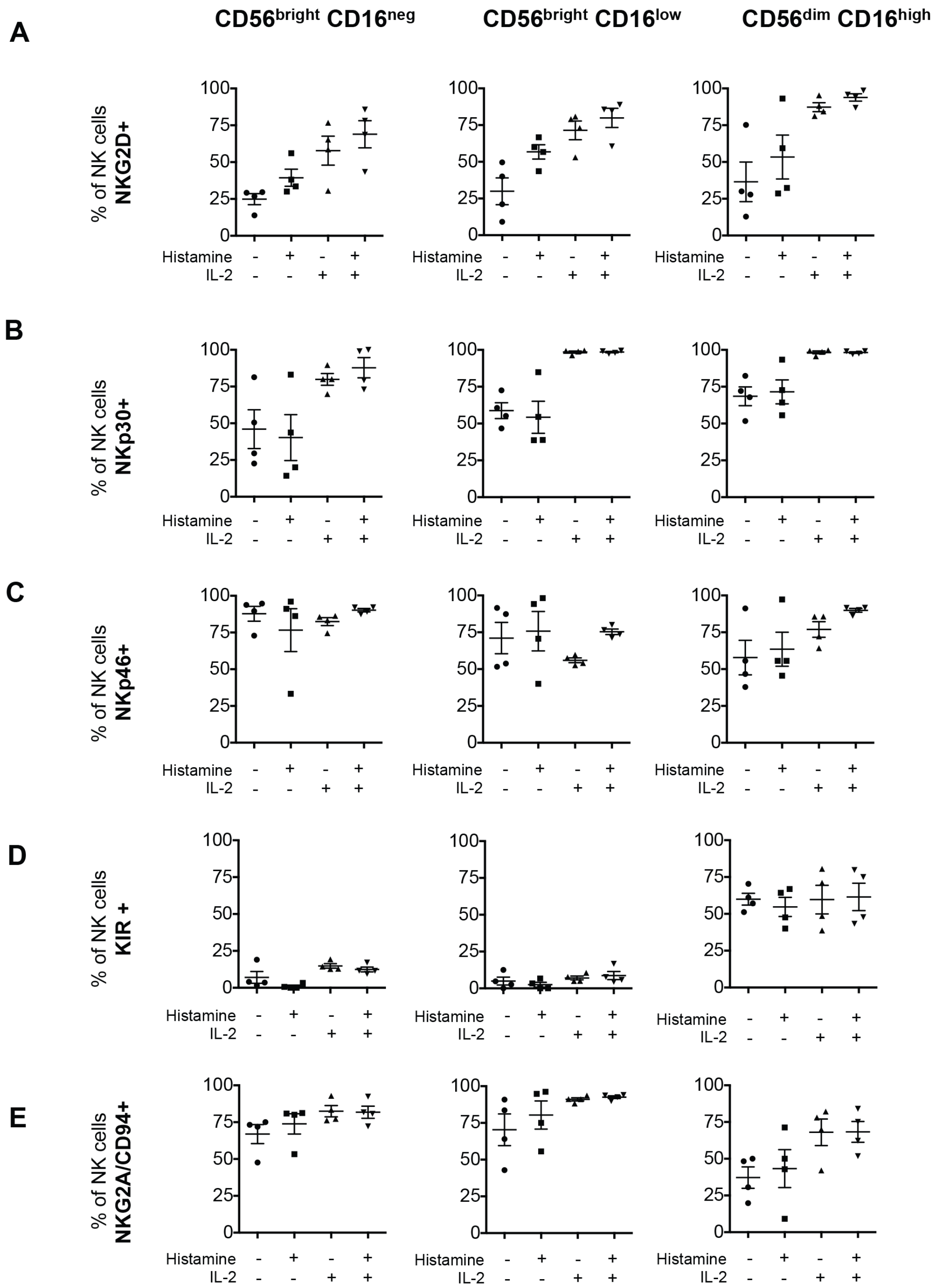

Supplementary Figure S3: *In vitro* effects of HDC and IL-2 on the expression of NK cell receptors in single NK cell subsets. PBMC from 4 healthy donors were stimulated with HDC (10^{-5} M), IL-2 (500 UI/ml) or a combination of both for 6 days. Percentages of cells expressing the receptors NKG2D, NKp30, NKp46, KIR and NKG2A. were analyzed on the $CD56^{bright}CD16^{neg}$, $CD56^{bright}CD16^{low}$ and $CD56^{dim}CD16^{high}$ subsets. The percentage of cells positive for the specific markers was determined as the number of positively stained cells minus the number of cells stained with an isotype-matched negative control antibody.

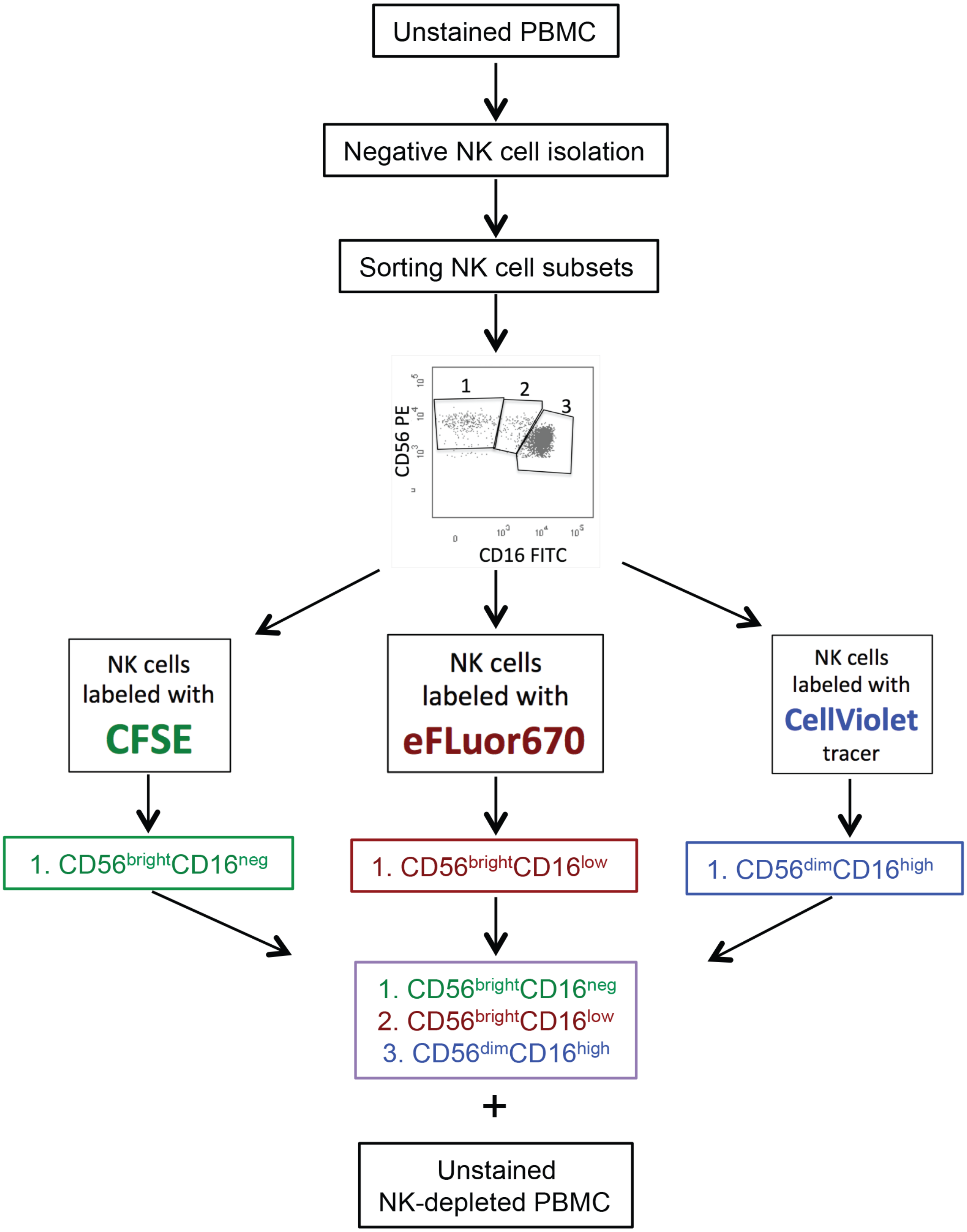

Supplementary Figure S4: Polychromatic and multifunctional tracing method. NK cells were isolated from PBMC of healthy donors by negative selection, stained with anti-CD56-PE and anti-CD16-FITC and sorted by preparative flow cytometry in a FACSAria. The three subsets were then individually labelled with either of the three cell tracer dyes CFSE ($CD56^{bright}CD16^{neg}$), eFluor670 ($CD56^{bright}CD16^{low}$) or CellTrace Violet ($CD56^{dim}CD16^{high}$) as described in Methods. The differentially labelled subsets were then recombined and a complete PBMC fraction reconstituted by adding the NK cell depleted fraction obtained during negative selection of the NK cells. The reconstituted PBMC fraction was then cultured for the respective experiments.
