# Supplementary material for: Maintenance therapy with histamine plus IL-2 induces a striking expansion of two CD56bright NK cell subpopulations in patients with acute myeloid leukemia and supports their activation
Source: Oncotarget. 2016 Jun 21;7(29):46466–81. doi: 10.18632/oncotarget.10191 (PMC5216810; doi:10.18632/oncotarget.10191)
Supplement: Supplementary file 2 [file oncotarget-07-46466-s002.docx]

**1. Supplementary tables**

|  | **Code** | **Sex** | **Age** |  | **Code** | **Sex** | **Age** | **Diagnose** |
| --- | --- | --- | --- | --- | --- | --- | --- | --- |
|  | **Healthy Donors** | | |  | **Histamine+IL2 AML patients** | | | |
|  | HD001 | F | 68 |  | HIL2001 | F | 21 | AML; FAB M4 |
|  | HD002 | M | 33 |  | HIL2002 | M | 37 | AML; FAB M2 |
|  | HD003 | M | 66 |  | HIL2003 | M | 59 | AML; FAB M5a |
|  | HD004 | F | 67 |  | HIL2004 | M | 50 | AML; FAB M5b |
|  | HD005 | F | 81 |  | HIL2005 | M | 55 | AML; FAB M4-eo with inv16 |
|  | HD006 | M | 83 |  | HIL2006 | M | 69 | AML; FAB M0 |
|  | HD007 | F | 23 |  | HIL2007 | F | 58 | AML; FAB M4-eo with inv16 |
|  | HD008 | M | 58 |  | HIL2008 | F | 28 | AML; FAB M1 |
|  | HD009 | F | 46 |  | HIL2009 | M | 34 | AML; FAB M4 |
|  | HD010 | F | 60 |  |  |  |  |  |
|  | HD011 | F | 58 |  |  |  |  |  |
|  | HD012 | F | 68 |  | **Untreated AML patients** | | | |
|  | HD013 | F | 78 |  | UNT001 | F | 74 | Secondary AML |
|  | HD014 | F | 36 |  | UNT002 | F | 83 | Secondary AML |
|  | HD015 | M | 61 |  | UNT003 | F | 62 | AML; FAB 1 AML |
|  | HD016 | M | 44 |  | UNT004 | M | 56 | AML FAB M2 |
|  | HD017 | M | 51 |  | UNT005 | F | 63 | AML; FAB M0 |
|  | HD018 | M | 43 |  | UNT006 | M | 72 | Secondary AML |
|  | HD019 | F | 27 |  | UNT007 | M | 69 | Secondary AML |
|  | HD020 | M | 30 |  | UNT008 | F | 37 | de novo AML, FAB M3v |
|  | HD021 | M | 75 |  | UNT009 | F | 67 | de novo AML; FAB M5a |
|  | HD022 | M | 36 |  | UNT010 | F | 23 | de novo AML; FAB M4 |
|  | HD023 | F | 27 |  | UNT011 | M | 75 | de novo AML; FAB M5 |
|  | HD024 | F | 34 |  |  |  |  |  |
|  | HD025 | M | 36 |  | **AML after Chemotherapy** | | | |
|  | HD026 | F | 27 |  | CHE001 | M | 44 | AML; FAB M2 |
|  | HD027 | M | 34 |  | CHE002 | F | 28 | AML; FAB M1 |
|  | HD028 | M | 30 |  | CHE003 | F | 72 | AML; FAB M1 |
|  | HD029 | F | 29 |  | CHE004 | M | 33 | AML FAB M4 |
|  | HD030 | F | 29 |  | CHE005 | M | 53 | AML with skin infiltration |
|  | HD031 | F | 24 |  | CHE006 | M | 57 | Secondary AML |
|  | HD032 | F | 46 |  | CHE007 | M | 64 | Secondary AML |
|  | HD033 | M | 27 |  | CHE008 | M | 38 | AML; FAB M2 |
|  | HD034 | M | 26 |  | CHE009 | M | 54 | AML; FAB M4 |
|  | HD035 | F | 25 |  |  |  |  |  |
|  | HD036 | F | 26 |  |  |  |  |  |
|  | HD037 | F | 31 |  |  |  |  |  |
|  | HD038 | M | 28 |  |  |  |  |  |
|  | HD039 | M | 64 |  |  |  |  |  |
|  | HD040 | F | 28 |  |  |  |  |  |
|  | HD041 | M | 34 |  |  |  |  |  |
|  | HD042 | M | 38 |  |  |  |  |  |
|  | HD043 | F | 68 |  |  |  |  |  |
|  | HD044 | M | 26 |  |  |  |  |  |
|  | HD045 | M | 41 |  |  |  |  |  |
|  | HD046 | F | 45 |  |  |  |  |  |
|  | HD047 | F | 29 |  |  |  |  |  |
|  | HD048 | M | 35 |  |  |  |  |  |

**Suppl. Table 1: List of AML patients and control healthy donors**

Compilation of the 11 untreated AML patients (average 62 years, range 23-83 years), 9 AML patients after chemotherapy (average 49 years, range 28-72 years), 9 AML patients treated in addition with HDC plus IL-2 (average 47, range 21-69 years) and the 48 healthy donors (average 43 years, range 23-83 years) from whom blood samples were obtained.
